# Changes of health related quality of life during pregnancy based on pregnancy context: a prospective study

**DOI:** 10.1186/s13690-022-00802-9

**Published:** 2022-01-21

**Authors:** Ashraf Kazemi, Aazam Dadkhah, Fatemeh Torabi

**Affiliations:** 1 Nursing and Midwifery Care Research Center, School of Nursing and Midwifery, Isfahan University of Medical Sciences, Isfahan, Iran; 2 Reproductive Health Department, School of Nursing and Midwifery, Isfahan University of Medical Sciences, Hezarjerib AV, Isfahan, Iran; 3 Student Research Committee, School of Nursing & Midwifery, Isfahan University of Medical Sciences, Isfahan, Iran

**Keywords:** Pregnancy, Health Related Quality of Life, Low risk pregnancy, Pregnancy, Family Planning, Unwanted pregnancy

## Abstract

**Background:**

The significance of planned pregnancy is an accepted principle for improving the health of pregnant women; and quality of life, as one of the important indicators of women’s health, is reduced in high-risk pregnancies. The aim of this research was to investigate the changes in the health related quality of life (HRQL) in low risk pregnancies in different groups based on pregnancy context.

**Methods:**

The present study was a prospective study conducted on 250 pregnant women divided into three groups of women with planned pregnancy, unplanned/wanted pregnancy and unwanted pregnancy. Then, using WHOQOL-26 questionnaire, the quality of life of these women was measured in physical, psychological, social and environmental dimensions at the beginning of pregnancy as well as at the end of the first, second and third trimesters.

**Results:**

Based on the results, the mean score of environmental-HRQL in women with unwanted pregnancy was significantly lower than the other two groups. All dimensions on HRQL were influenced by time and group. However, changes in the physical, psychological and social dimensions of HRQL varied within the groups. Physical- HRQL changes were different within the groups. The intergroup effect on environmental dimension of quality of life changes was significant.

**Conclusions:**

It was observed in this study that HRQL in the women with unwanted pregnancy was lower than the women with planned pregnancy and those with unplanned /wanted pregnancy. Moreover, increase in gestational age would lower quality of life, but this decline had a similar pattern in different groups.

## Background

Pregnancy is a complex period in women’s lives. It is associated with significant physical and psychological changes that play an important role in balancing the functions of different systems in women’s body with regard to the needs of the fetus. Although necessary for the survival of the fetus and the adaptation of the mother’s body to new conditions, these changes make pregnant women vulnerable to physical and mental problems [1]. Additionally, these changes in various stages of pregnancy are associated with common complaints such as morning sickness, low back pain [2], movement restriction, Pelvic Girdle Pain [3] and sleep disorder [4]. Although these transient problems can be tolerated by most pregnant women and do not seriously threaten their health, they reduce the quality of life of these women during pregnancy [1].

Quality of life is an important criterion in evaluating development indicators. While health is defined as complete physical, psychological, and social well-being, quality of life is a mental perception of health [5] affected by illnesses as well as physical [6] and mental [7]. High-risk and complicated pregnancies are among the factors affecting the decline of the HRQL in pregnant women [8]. Nonetheless, change in HRQL in low risk pregnancies have received less attention. Moreover, due to the mental nature of quality of life, it can be influenced by different aspects of life. So that pregnancy complications such as hyperemesis have increased drastically after unwanted and unplanned pregnancies [9]. There are also studies reporting the low trend in HRQL following an unplanned pregnancy [10]. Although physical and psychological changes of pregnancy and the complications caused by them occur for most women, their impact on quality of life may be influenced by women’s preparation and planning for pregnancy.

Planned pregnancy is one of the major goals of reproductive health whose desirability is an accepted principle in family planning as well as maternal and child health [4]. Denial of pregnancy following an unplanned pregnancy may reduce a person’s psychological tolerance to pregnancy changes.

As the results of a qualitative study showed, couples defined planned pregnancy as adaptation to the conditions of having a child. Common problems in pregnancy such as pelvic girdle pain [11] and low back pain [12] and body dissatisfaction [13] may reduce the quality of life of pregnant women. Accordingly, family planning for childbearing provides the couples with the necessary readiness to accept pregnancy, and may reduce the negative effects of pregnancy on quality of life. However, it should be noted that even unplanned pregnancy can also be associated with pregnancy acceptance [14] and lessen the negative effects of unplanned pregnancy on the HRQL of pregnant women. In addition, the impact of unplanned and unwanted pregnancies on HRQL can be subject to the cultural beliefs and values governing different societies.

The impossibility of legal abortion because of religious values in some societies increases the likelihood of continuing unplanned and unwanted pregnancies. As such, understanding the changes in quality of life during unplanned and unwanted pregnancies seems to be essential in these societies. Accordingly, this study investigated change in HRQL of pregnant women in terms of pregnancy context (PC).

## Method

This prospective study, approved by the Ethics Committee of Isfahan University of Medical Sciences, was conducted between April 2017 and January 2018 in Isfahan-Iran. Taking into account the 95% confidence level, the level of significance (α), 0.05 (Zα/2 = 1.96), the power 80% and the maximum acceptable error in estimating the comparison of principal variables 0.7 and the variance of depressive changes (∂:^2^=7) the sample size was calculated 250 women. The study was conducted on 250 women with gestational age of 6-9 weeks who referred to ten Health Centers in Isfahan to start prenatal care. Based on the weekly number of clients in each center, between 20 and 30 samples were considered. Inclusion criteria were aged between 18 and 35 years, no history of complications in previous pregnancies, no systemic disease in current pregnancy, and no known pre-pregnancy psychological disorder. Exclusion criteria were including twin or multiple pregnancy diagnosis and abortion in the first trimester, pregnancy complications such as preeclampsia and second trimester pregnancy bleeding, and stressful events such as death of relatives, economic bankruptcy and suchlike.

The centers were selected from two networks in Isfahan using stratified random cluster sampling. and the pregnant women were selected by convenience sampling method and all eligible pregnant women were invited to participate in the study and, after obtaining informed consent, their demographic data were recorded.

To determine PC, intention and wantedness for pregnancy were asked about using interview and those who were pregnant with a pre-planned program were put into the planned pregnancy group, those who were pregnant without a predetermined program but wanted the baby, were put into the unplanned/wanted group and, finally, those who became pregnant without a predetermined program and did not want the baby were placed in the unwanted pregnancy group [14].

The subjects’ HRQL was measured using WHO Quality of Life-BREF (WHOQL-26) at 11-12 (T1), 24-25 (T2) and 33-35 weeks (T3) of pregnancy (4) .

The validated WHOQL-26 is a self-reported survey that consists of 26 questions regarding quality of life and is divided into four domains physical health (7 items), psychological health (6 items), social relationships (3 items), and environmental health (8 items). This questionnaire was performed on a 5-point Likert scale (strongly agree: 5 score to strongly disagree: 1 score). The scores of each domain and also total score were transformed linearly to a 0–100-scale [15]. The questionnaires were completed by women.

### Statistical analysis

Data analysis was performed using IBM SPSS 19 software (SPSS Inc., Chicago, IL, USA). Normal distribution assumption was tested by the Kolmogorov-Smirnov test. Since, the HRQL and its domains score were normally distributed; parametric statistics were applied for the data analysis. Baseline characterizes were compared between three groups of women, based on PC using analysis of variance (for age), Chi-square (for employment status) and Mann–Whitney U test (for education and gravity) The mean scores’ differences of dependent variables in T1, T2 and T3 between three groups of women, based on PC were evaluated using analysis of covariance (ANCOVA), controlling for baseline data and independent variables (group as fixed parameter and mean scores of the HRQL and its domains at T1 as covariate). The variables women’s age, educational level, gravidity and employment status were considered as potential confounders and were compared in three groups of PC. Variables that were different between the three groups were entered in the models. Repeated measure analysis of variance (RMANOVA) was used to assess the changes in HRQL during the study follow-ups. Also women education as potential confounder was entered in the models.

## Results

**Fig. 1:**
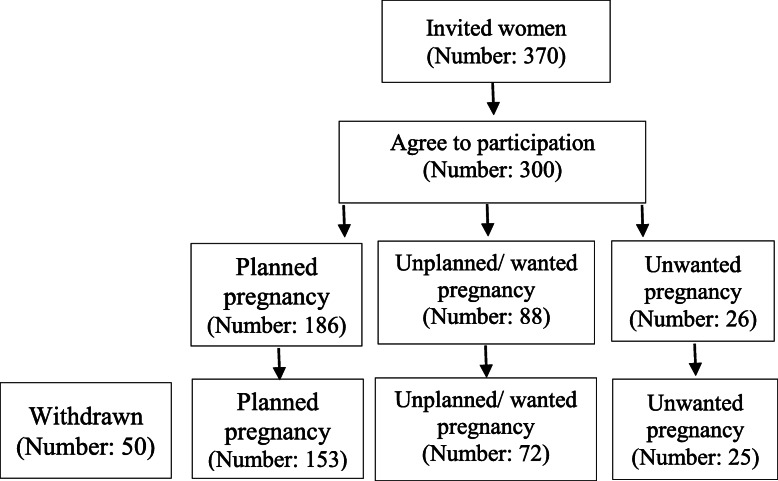
Study flowchart

Of the 370 women invited to participate in the study, 300 accepted to take part in it. 29 pregnant women because of abortion and 21 others because of pregnancy complications such as preeclampsia, bleeding in the second trimester, and preterm labor pain were excluded from the study. Then, the data of 250 pregnant women aged between 18 and 35 years old were analyzed. Among these women, 153 women (61.2%) had planned pregnancy, 72 women (28.8%) unplanned/ wanted pregnancy, and 25 women (10%) had unwanted pregnancy (Fig. 1).

Based on the results, the three groups were not different in terms of age, gravidity, employment status. However, the frequency of under-diploma education level in the unwanted and unplanned pregnancy groups was higher than that of the planned pregnancy group (Table 1). Therefore. women’s education was entered in the models as covariant variable.

**Table 1:** The baseline characteristics of the subjects

	Mean (standard deviation) or Number (%)			Sig
**Groups**	Wanted/ Planned	Wanted /Unplanned	Unwanted	
Number	153	72	25	
**Age**	28.7 (3.9)	29.0 (4.6)	28.6 (3.7)	0.84
**Gravity**	1.5 (0.8)	1.39 (0.6)	1.83 (0.9)	0.09
**Education(%)**				0.001
High school	13 (8.5)	8 (11.1)	3 (12.0)	
Diploma	69 (45.1)	23 (31.9)	13 (52.0)	
Academic	71 (46.4)	41 (56.9)	9 (36.0)	
**Occupation (%)**				0.10
Employed	22 (8.8)	9 (5.9)	9 (12.5)	

_Rials (R): Eeach dollar equal to 45000 Rails at the of data gendering time in this study_

The mean score of the physical, psychological and environmental HRQL at T1 in women with unwanted pregnancy were lower than the other groups. The results of the ANCOVA test with controlling education level and dimensions of HRQ at T1, showed that, adjusted to the education level, the mean score of all dimensions of HRQL at T2 and T3 were different between groups (Table 2). The mean score of the dimensions of HRQ in planned/ wanted and unplanned/ wanted group decreased at T2 and T3; however, these variables in the women with unwanted pregnancy were lower and more stable.

**Table 2:** Comparison of health related quality of life domains mean scores between the groups

	Time 1			Time 2			Statistical results			Time 3			Statistical results		
	Mean (SD)			Mean (SD)						Mean (SD)					
**HQOL**	G1	G2	G3	G1	G2	G3	**F**^**h**^	**P**^**h**^	**ηp**^**2h**^	G1	G2	G3	**F**^**h**^	**P**^**h**^	**ηp**^**2h**^
**Physical**	27.3 (3.9)	27.1 (3.9)	23.3 (3.5)	23.7 (5.3)	23.4 (4.5)	24.0 (6.1)	95.0	<0.001	0.29	24.3 (3.7)	24.2 (4.3)	23.8 (3.8)	146.8	<0.001	0.38
**Psychological**	23.3 (3.1)	22.6 (3.3)	21.3 (3.7)	20.5 (4.1)	20.1 (4.0)	20.0 (4.1)	132.5	<0.001	0.35	21.1 (3.5)	20.3 (3.6)	20.0 (3.6)	152.5	<0.001	0.39
**Social**	12.1 (1.5)	12.0 (1.3)	11.7 (1.4)	10.6 (2.1)	10.6 (2.2)	10.8 (1.9)	61.8	<0.001	0.20	11.1 (2.1)	10.7 (2.0)	10.9 (1.9)	79.9	<0.001	0.25
**Environmental**	30.4 (3.5)	29.1 (3.3)	28.9 (3.8)	28.9 (3.9)	27.5 (4.4)	27.5 (3.2)	248.2	<0.001	0.50	29.9 (3.9)	27.8 (3.8)	28.1 (4.3)	233.4	<0.001	0.49

Abbreviations: G1: Planned pregnancy; G2: Unplanned/ Wanted pregnancy; G3: Unplanned/ unwanted pregnancy; HQOL: health related quality of life; SD = Standard deviation, ηp2= partial eta-squared

The results of RMANOVA showed that the interaction effect of time and education level on physical, psychological and environmental HRQL and also, interaction effect of time and PC on physical HRQL were significant (Table 3). The effect of education (F=13.7, *P*<.001) and PC (F=5.07, *P*=.007) on mean score of the environmental HRQL were significant (tests of between subjects’ effects).

**Table 3:** Differences in the HRQOL at the three assessing times (repeated measure analysis of variance)

	Mean (standard deviation)			Time/ Group		Time/ Education	
	Time 1	Time 2	Time 3	F^a^	Sig^a^	F^a^	Sig^a^
Total HRQOL	91.8 (10.2)	83.2 (12.8)	61.51 (16.0)	1.1	0.33	4.7	0.009
Physical HRQOL	27.0 (3.9)	23.9 (4.9)	24.1 (4.5)	2.7	0.02	6.3	0.002
Psychological HRQOL	12.0 (1.5)	10.6 (2.1)	20.6 (3.6)	1.2	0.37	8.7	<0.001
Social HRQOL	22.9 (3.3)	20.3 (4.1)	11.0 (2.1)	1.1	0.55	2.1	0.08
Environmental HRQOL	29.8 (3.6)	28.3 (4.1)	28.6 (3.9)	0.2	0.97	3.5	0.03

**Abbreviations**: HRQOL: Health related Quality of Life; ^a^ test of between subject effect; ns: non-significant

## Discussion

This study aimed at investigating the change in HRQL during uncomplicated pregnancies with regard to PC. To this end, the quality of life changes were compared between three groups of pregnancy context; women with planned, unplanned/wanted and unplanned/unwanted pregnancies. The results showed that HRQL had a decreasing from the beginning of pregnancy to the third trimester. Although women with planned pregnancies have a higher quality of life with the onset of pregnancy, they experience similar changes compared to the other two groups.

Comparing the background characteristics of the groups, it was shown that the frequency of under-diploma education level in women with planned pregnancy was lower than the other two groups. This finding corroborates the results of other studies indicating that women’s education is a determining factor in family planning [16, 17]. Nevertheless, unlike other studies [18–20], it was not a determining factor in quality of life. Additionally, contrary to the results of other studies [21, 22], the results of the present study demonstrated that number of pregnancies, and age were not related to planning for pregnancy.

Evaluation of change in HRQL over four periods of time confirmed the effect of time on the total quality of life score and its different dimensions. So that the HRQL score decreased during the first trimester, then, remained stable during the second trimester and, finally, began to decrease again after the second and during the third trimesters. This change was consistent with the situation of pregnant women during different pregnancy periods. Common problems in the 1th trimester, such as morning sickness [23], lead to lower HRQL in this trimester.

In the second trimester, decrease in this sickness often results in fewer problems for women, explaining how quality of life does not change during the second trimester. Increased prevalence of pregnancy problems following an increase in abdominal enlargement, restriction of movement and sleep disorder during this period [24] is also associated with decreased quality of life. Decreased quality of life associated with the increase of gestational age has been reported previously [17], and the present study attempted to show that this change occurs even during uncomplicated pregnancies Als, a study on Chinese women pregnant showed an increase in quality of life in the second trimester of pregnancy. In this study, the quality of life in the third trimester had been reported at the lowest level [25].

In line with the main objectives of this research, the results suggested that, from the outset of pregnancy, women in the unwanted pregnancy group reported lower quality of life compared to the other two groups; moreover, throughout the pregnancy, their quality of life in the environmental dimension was lower than the other two groups. Changes in overall quality of life and quality of life in physical, psychological and social dimensions followed the same pattern in all three groups. This finding suggests that, except for the environmental dimension of quality of life, other aspects are less affected by unwanted pregnancy during pregnancy; while this factor (unwanted pregnancy) has an ever-lasting impact on the environmental dimension of HRQL.

As previous studies have shown, unwanted and unplanned pregnancies can reduce quality of life in women [20]. Nonetheless, no difference was observed in the present study in the quality of life of the women with planned pregnancy and those with unplanned/wanted pregnancy. According to this finding, the wantedness of the pregnancy in the women of the study, even if not based on previous planning, does not have an adverse effect on the quality of life of these women. By contrast, unwanted pregnancy can reduce the quality of life of pregnant women.

Providing physical, psychological, social and environmental facilities is one of the conditions considered by people judging the timeliness of pregnancy. Put differently, a pregnancy is considered timely when it occurs in an appropriate condition. The environmental dimension of quality of life refers to the availability of environmental and supportive facilities [4] and, it should be noted, that differences between groups are expected at the beginning of pregnancy. But it was observed unexpectedly within the first trimester, that women with planned pregnancies experienced more decrease in the environmental dimension than women with unplanned/wanted pregnancies.

Decreased environmental quality of life during the second trimester for the women with planned and wanted pregnancies is maybe due to an incorrect estimation of conditions for planning the pregnancy. Furthermore, although perceived social support has not been measured in this study, decreased environmental quality of life in this women suggests that the available support systems have failed to decrease the problems of women during the first trimester.

One of the limitations of the present study, that needs to be taken into account in interpreting the results, is that poor pre-pregnancy quality of life can be a reason for women’s unwillingness to become pregnant. Therefore, to identify the causal pathway between PC and quality of life, the pre-pregnancy quality of life of these women needs to be assessed. In addition, the low number of women with unwanted pregnancies is another limitation of the study that may affect the results of the study.

## Conclusions

The results of this study showed that the increase of gestational age can reduce the quality of life in uncomplicated pregnancies. It was also shown that based on PC there is a similar pattern of change in physical, psychological and social dimensions of quality of life in different groups of women. However, the environmental dimension of quality of life in women with unwanted pregnancies is lower than other groups and, hence, needs to be taken into account in planning pregnancy cares for these women.

## Data Availability

Data and material are available on request from the corresponding author.

## Abbreviations

HRQL: health related quality of life
PC: pregnancy context
T: Time
ANCOVA: analysis of covariance
RMANOVA: repeated measures analysis of variance
G: group
ηp2: partial eta-squared
